# Tourists’ Food Involvement, Place Attachment, and Destination Loyalty: The Moderating Role of Lifestyle

**DOI:** 10.3390/bs13080629

**Published:** 2023-07-28

**Authors:** Jingru Chen, Fu-Chieh Hsu, Libo Yan, Hoffer M. Lee, Yuqing Zhang

**Affiliations:** 1 Faculty of Hospitality and Tourism Management, Macau University of Science and Technology, Macau, China; limeili@xpc.edu.cn (J.C.); 2109853gbt30004@student.must.edu.mo (Y.Z.); 2 Department of Cultural Tourism, National United University, Miaoli City 360302, Taiwan; fchsu@nuu.edu.tw; 3 Centre for Gaming and Tourism Studies, Macao Polytechnic University, Macau, China; lbyan@mpu.edu.mo

**Keywords:** food tourism, food involvement, place attachment, destination loyalty, lifestyle

## Abstract

Destination food has been increasingly appealing to tourists within adjacent markets. This trend has been widely recognised by destination marketers; however, scholars have paid limited attention to tourists’ psychological and behavioural responses to destination food. Taking Shunde as the research site, using a questionnaire-based survey method equipped with the techniques of measurement modelling, path analysis, principal component analysis, and cluster analysis, this study explores how tourists’ food involvement affects their place attachment and destination loyalty, as well as the differentiation of tourists with different lifestyles. The results show that food involvement significantly affects place attachment and destination loyalty. These relationships are differentiated by tourists’ lifestyles. This study contributes to the psychological research of tourist behaviours and provides insights into destination marketing in the context of food tourism.

## 1. Introduction

At present, an increasing number of people are travelling to taste local delicacies in different countries [1], and food plays an essential role in tourists’ choices of tourist destinations [2]. The attractiveness of food to tourists and the importance of food to destinations have been recognised by destination marketers [3]. There is a limited understanding in the literature of how destination food affects food tourists’ psychological states and behaviours. 

In the highly competitive tourism market, an effective marketing strategy is one of the critical factors to success. Effective marketing should be based on a sufficient understanding of the tourists. In light of this aspect, numerous related studies have investigated tourists’ experience, satisfaction, and loyalty [4,5,6]. In the field of food tourism research, food involvement has been one of the preferred entry points for research related to food tourism [7]. Loyalty has always been one of the hotspots in the academic circle of tourism and has also become an essential index for many tourism managers and researchers to evaluate the tourism market, resources, and products. Studying the antecedent variables of tourist loyalty has always been the core research topic in the literature [8]. Unfortunately, the relationship between food involvement and loyalty has been under-investigated as well as the relationship between food involvement and place attachment. From the current research results, satisfaction, tourism motivation, service quality, perceived value, and tourism destination image can be determined as the five essential antecedent variables that affect tourist loyalty [8]. In addition to the abovementioned variables, existing studies have indicated that place attachment can also have a significant positive impact on the loyalty factor [9]. However, the mediating effect of place attachment on the relationship between food involvement and loyalty remains unknown.

McCleary [10] highlighted that lifestyle segmentation is a powerful marketing tool in the tourism industry because it does not only consider demographic factors, but also focuses on tourists’ values, attitudes, opinions, and interests, which places the focus on the tourist’s identity. Compared with the demographic characteristic segmentation method, lifestyle is a market segmentation method that presents significant advantages and, in turn, can help to thoroughly explore the psychological traits of tourists. Levitt et al. [11] conducted a study to identify different food tourist groups based on their food involvement and motivation to determine whether the potential food tourism groups differ in attitudes, a willingness to consume local food, or food tourism planning behaviours. Nevertheless, studies have yet to investigate whether there are differences present in the emotional and behavioural intentions of different food tourism groups towards food tourism destinations during travel. 

The primary purpose of this study is to investigate the link between tourists’ food involvement and destination loyalty, as well the indirect relationship through place attachment. Moreover, this study investigates whether tourists’ lifestyles moderate the relationships between food involvement, place attachment, and loyalty. Therefore, the research questions of this study are as follows: (1) How does food involvement affect tourists’ place attachment? (2) How does food involvement affect tourists’ loyalty? (3) How does food involvement affect tourists’ loyalty through their place attachment? (4) How does lifestyle moderate the relationship between food involvement and tourists’ place attachment as well as the relationship between place attachment and tourists’ loyalty? Shunde, China was selected as the location for a case study to fulfil the research objectives. It was awarded the “World Food Capital” by UNESCO in 2014.

## 2. Review of the Literature

### 2.1. Theoretical Background

The stimulus–organism–response (S-O-R) theory is the underpinning theory to explain the conceptual framework in this study. It is a framework from a psychological perspective that is used to delineate the process of individual behavioural formation and response. This theory identifies the following three primary components as the essential constituents: stimulus, organism, and response. Specifically, the stimulus comprises the external and environmental cues that exert an influence on the individual, which also functions as the triggering [12]. The organism, on the other hand, is the internal state, either emotional or cognitive, which is provoked and affected by the external stimulus [13]. Finally, the individuals’ internal states are the antecedents forming their response, including decision making and the behavioural outcomes [14]. Therefore, external factors and environmental cues trigger an individual’s internal feelings and emotions, which eventually guide their behaviour.

In tourism and hospitality research, the S-O-R theory is valuable in depicting tourists’ and customers’ behaviour and providing clear insight into the formation process. For example, Hsu, Agyeiwaah, and Chen [15] investigated the food festival attendees regarding their loyalty behaviour based on the S-O-R theory. The results show that the S-O-R framework provides a valuable interpretation for loyalty behaviour formation. Wu, Wong, and Lin [16] successfully explained tourists’ behavioural intention by applying the S-O-R theory and identified that online atmospheric cues are an important influence. Similarly, Sohaib, Wang, Iqbal, and Han [17] concluded that the S-O-R theory serves as an adequate theoretical framework for clarifying the influence of nature-based solutions on green brand attitudes among hotel guests.

The S-O-R theory is ideal for this study as it can elucidate the formation process of tourists’ loyalty to a destination. Food involvement, being a trait and characteristic, significantly influences tourists’ perceptions and attitudes towards their food experience in the travel destination [18]. Consequently, it can be considered an essential element and feature of the stimulus [19]. In this study, food involvement influences tourists’ reactions to the food-related environmental cues in the travel destination, serving as the stimulus that would elicit emotional states in tourists. Furthermore, place attachment emphasizes the emotional bond that individuals form with a specific place or environment [20]. In this research, place attachment represents an internal feeling characterized by a high level of affective components, functioning as the emotional state of tourists. Lastly, destination loyalty is a typical response to behavioural outcomes, which was identified in various studies [21,22]. 

### 2.2. Food Tourism and Food Tourists

Food and tourism have been compelling areas of tourism research over the past three decades since the exploration conducted by Belisle [23] on this topic. Food strongly influences travellers’ decisions when choosing a destination for a vacation [24]. Food consumption is one of the critical travel experiences and motivations [25]. Conceptually, food-based tourism or food tourism is characterised by food-related tourism, including the tasting of food, becoming familiar with the food production process [26], and experiencing local food culture [27]. Specifically, food tourism includes visits to food producers, food festivals, restaurants, and specific locations [28]. This type of tourism has also been labelled as culinary tourism [29], gastronomy tourism [30], and tasting tourism [31]. A certain degree of conceptual overlap exists between the different types of food tourism; however, subtle discrepancies can be identified. Food tourism focuses on food as the primary motivation in travel; culinary tourism focuses on the preparation of food and the process of cooking; gastronomy tourism focuses on the study of gastronomy; and tasting tourism focuses on the consumption of food and drink. In the context of this study, tourists visited Shunde to taste the local specialities and visit well-known local restaurants. The tourism phenomenon this study investigates is in line with the concept of food tourism. 

Ignatov [32] first proposed the concept of “food tourists”, referring to tourists who have travelled to a destination and participated in various activities. These activities include browsing or buying gourmet ingredients at retail stores or farms, tasting food at local restaurants, dining at internationally renowned restaurants, studying in culinary schools, or staying in gourmet restaurants with accommodation facilities. Wolfe [33] further suggested three scenarios specific to food tourists visiting a new restaurant within a one- or two-hour drive, visiting foreign grocery stores or food markets during overseas trips, and having a vacation associated with food festivals or seasonal fruit or vegetable plantations. Robinson and Getz [34] proposed a more segmented concept of food tourists called food fanatics. This group of people are interested in “eating” and “talking” about food. They care about where the food comes from, the preparation process, and even the raw materials used to achieve a more advanced level of food appreciation. In this study, food tourists are represented by those who travel to a destination to taste the local specialities and eat in well-known local gourmet restaurants. Robinson and Getz [34] proposed that destinations need to better understand tourists’ involvement in food to attract foodies. Food tourists are composed of different levels of food involvement and motivations [35].

### 2.3. Food Involvement

Involvement refers to the degree of psychological connection [36], the perception of personal relevance [37], and the degree to which a person is engaged in an object, activity, place, or experience [38]. Zaichkowsky [39] defined involvement as a state of motivation, arousal, or interest, and the degree to which an individual perceives the importance of something based on their own needs, interests, and values. The concept of involvement first received attention in the field of food intake in the early 2000s [40,41]. Bell and Marshall [38] first defined food involvement as “the level of importance of food in a person’s life” and pointed out that the level of food involvement may vary from individual to individual. They believe that cravings for new food flavours may increase food’s importance in food consumers’ lives. By combining scholars’ definitions of involvement in the field of tourism and the definition of food involvement proposed by Bell and Marshall [37], we defined tourists’ food involvement as the importance of food for tourists during their journeys, and the perception of importance reflects their own needs, interests, and values.

A scale to measure food involvement was derived from the personal involvement scale proposed by Zaichkowsky [39]. In this scale, involvement can be conceptualised as the degree to which a person associates themself with an activity or product through attractive, powerful, interesting, valuable, exciting, thrilling, desirable, wanted, and meaningful aspects to explore tourists’ food involvement [42]. Another scale specific to food involvement was proposed by Bell and Marshall [40], which measures food involvement stages, including food access, preparation, cooking, eating, and handling-related activities. Kim et al. [43] utilised the abovementioned scale to study the food involvement of Korean kimchi festival tourists. Caber et al. [7] also used this scale when studying the impact of food involvement on local food consumption. However, for the purpose of this study, the items related to preparation and processing were removed, and the items related to diet, acquisition, and cooking were retained. As a result, in this study, the food involvement scale proposed by Bell and Marshall [40] was believed to be more pertinent, and the questions presented were more context specific. Therefore, a modified scale adopted from Bell and Marshall [40] was used for this study.

### 2.4. Place Attachment

According to Stokols and Shumaker [44], place attachment is the degree to which an individual functionally associates themself with a place. Place attachment reflects an emotional bonding activity between an individual and a specific spatial environment [45], or the emotional investment in a place [46]. Based on previous studies, we defined place attachment as tourists’ emotional sustenance and functional dependence on a destination.

Scholars disagree over the dimensions of place attachment. Place identity and place dependence are two dimensions widely adopted by place attachment studies in various contexts [47]. William and Vaske [48] adopted psychometric methods to design a place attachment scale to validate a two-dimensional conceptual framework (i.e., place identity and place dependence) for tourism destinations. Harmon et al. [49] added an independent structure of emotional attachment to the original model, which is considered to be an individual’s emotional attachment to a place, giving it meaning [50]. Other scholars believe that place attachment possesses additional dimensions, such as place affect and place social bonding [51,52]. Based on the research’s similarity and the model’s reliability, the place attachment model constructed by place dependence and place identity was adopted in this study.

Place identity refers to people’s identification with a place they consider unique [51] or a place that matches their own identity [53]. The tourism environment can express and affirm one’s own identity. Therefore, place identity can increase one’s sense of belonging to a tourist destination [54]. In this study, food lovers who travel to a widely recognised food city may affirm their identity as foodies and thus develop a sense of attachment to this place. 

Place dependence refers to a functional attachment to a place [55], which reflects the importance of the place in achieving one’s functional goals (or activities) [56]. This functional dependence is reflected in the physical characteristics of the destination or region [48]. Places that can meet multiple needs generate more profound and broader place dependencies for tourists than those that meet limited needs [44]. Simply put, when a person emotionally connects with, identifies with, or feels as though they belong to a place, an identity with the place is then developed. If the environment of a place can meet an individual’s functional needs, they will develop a dependence on the place.

### 2.5. Tourist Loyalty

Studying tourists’ loyalty is helpful for the marketing and management of a given destination [57], which is pivotal for the profit and sustainable development of the destination [58]. Loyalty is defined as repeat purchase behaviour characterised by repurchase intention, word-of-mouth communication, and recommendation intention (Lee et al. [59]). Oliver and Burke [60] highlighted that loyalty depends on customer satisfaction, which is considered to be the consumers’ judgment of goods and services. Travel loyalty has long been perceived as an extension of customer loyalty in the travel environment [61]. A destination can be regarded as a product and satisfy customer needs, or loyal tourists may revisit or recommend it to others [6]. 

The concept of loyalty can be understood via the following three dimensions: behavioural, attitudinal, and comprehensive loyalty. Behavioural loyalty refers to the behaviour of repeat purchases or the proportion of purchases, and this dimension focuses on outcomes. Attitudinal loyalty is “customers’ stated preferences, commitments, or purchase intentions” [62]. In tourism, attitudinal loyalty refers to the psychological outcomes of tourists, such as the intention to visit a particular destination repeatedly or recommend it to others. Comprehensive loyalty is a combination of loyalty-related attitudes and behaviours [63]. 

From the perspective of the tourist destination, revisiting and recommending it to others are thought to be related to behavioural loyalty [6]. This viewpoint is also supported by Opperman [64], who claims that the tourist experience at the destination is a product, and their loyalty can then be reflected in the behavioural intention to revisit the destination and recommend those experiences to friends and family. However, some scholars classify it as attitude loyalty because it presents a positive attitude towards the destination. This attitude strongly indicates an individual’s tendency to recommend destinations to other tourists, even when they do not revisit them at all [65]. To sum up, this study defines tourist loyalty as the comprehensive loyalty of willingness to revisit and present positive information, and it uses the two elements of intention to revisit and recommendation to relatives and friends to predict tourists’ loyalty to certain tourist destinations [6]. 

### 2.6. Lifestyle

The term “lifestyle” comes from the fields of psychology and sociology. It was first studied in depth in the 1960s by psychologist Alfred Adler, who emphasised the uniqueness of the individual, but also recognised the similarity between the individual and their way of life. Lazer [66] defined lifestyle as a systematic concept, attributing this particular pattern to the aggregation and development of people in society. In addition, lifestyle also emphasises social and cultural values and behaviours and is often used to describe the daily life of consumers [67]. Later, scholars proposed the concept of lifestyle as sociological, describing it as the way individuals allocate time and money, the way they display activities and things that interest them, and their views on various issues [68,69]. Hawkins et al. [70] regard lifestyle as an embodiment of self-concept, suggesting that lifestyle is shaped by past experiences, intrinsic characteristics, and the environment at the time. Plummer [71] believes that lifestyle can be divided into the following two concepts: the mode of lifestyle and the way of market segmentation based on lifestyle. In marketing management studies, it is crucial to understand customers’ needs through their lifestyles. Marketers can effectively communicate with and market to customers by knowing and understanding their lifestyles [71]. As a result, lifestyle is also considered an effective segmentation tool in marketing research and is widely adopted by marketers [63].

Plummer’s [71] AIO (Activities, Interests, and Opinions) scale is often utilised by researchers to measure people’s lifestyles [72,73]. Activities include work, hobbies, social activities, vacations, and community; interests include family, entertainment, food, and media; and opinions include education, culture, social issues, and business. Another scale, the Values and Lifestyle Survey (VALS) [74], incorporates the concept of value into the measurement tool and reinforces the integrity of the AIO scale in measuring lifestyle. It divides individuals into the following eight groups: thinkers, believers, innovators, achievers, strugglers, survivors, experiencers, and creators. While studies using the AIO or VALS methods yield insightful and meaningful data, they require extensive investigation and analysis and are operationally difficult [73,75], as these studies typically use 300 or more items [73]. Furthermore, Chinese consumers’ perceptions and behaviours are quite different from those in Western countries [76], and AIO and VALS may not be suitable for Chinese consumer research. Based on the complexity of the AIO and VALS scales and the particularity of China’s national conditions, this study uses a less-complicated scale validated in the Chinese context [77]. The scale was derived from the AIO-based scale developed by the authors of [78]. Tian [77] combined tourism motivation as an index and selected ten measurement indices, including health, family, sports, travel, work, adventure, culture, novelty, leisure time, and environmental awareness, and constructed a lifestyle measurement index. 

### 2.7. The Relationships among Food Involvement, Place Attachment, and Tourist Loyalty

Previous studies have examined the relationships among the three constructs in various contexts. For instance, Brown et al. [79] conducted a survey on tourists during the London Olympics and demonstrated that sports involvement positively impacts place attachment, thereby influencing tourists’ willingness to revisit a place. In a study by Hsu and Scott (2020) [18], it was suggested that food experience plays a crucial role in positively influencing place attachment in travel destinations. Similarly, Wang et al. [80] conducted a study on rural tourism and found that tourism involvement significantly influences place attachment. Furthermore, Chen [81] investigated the relationship between involvement and place attachment in film tourism and discovered that celebrity involvement affects tourists’ place attachment to the destination. Moreover, Plunkett et al. [82] confirmed that place identity and place dependency exert a significant influence on behavioural loyalty. Additionally, tourist involvement has been found to impact the perceived value of the destination experience [82]. Based on the preceding discussions, we proposed the following hypotheses:

**H1a.**  *Food involvement of tourists has a positive impact on place identity.*

**H1b.**  *Food involvement of tourists has a positive impact on place dependence.*

**H2.**  *Food involvement of tourists has a positive impact on loyalty.*

### 2.8. The Mediating Effect of Place Attachment

As previously discussed, the relationship between food involvement and place attachment is positive, and so is the relationship between place attachment and loyalty. Place attachment can lead to a positive evaluation of the destination experience [9] and thus increase tourists’ willingness to revisit the place [83]. A greater place attachment value can cause tourists to have a stronger sense of loyalty to the destination [56]. Tourism studies have revealed that both dimensions of place attachment (identity and dependence) positively impact tourists’ intention to recommend the destination to their friends or to revisit it [84,85]. Moreover, Wang et al. [80] investigated the mediating role of place attachment in the context of rural tourism. The results of their study reveal that place identity and place dependence mediate the relationship between involvement and loyalty. Similarly, the mediating role of place attachment was also confirmed by Nasir et al. [86]. Specifically, their study indicates that the relationship between destination attractiveness and loyalty is fully mediated by place attachment. The involvement–attachment–loyalty link in the tourism context might apply to special-interest tourism, namely, food tourism. Thus, we proposed the following hypotheses:

**H3a.**  *Place identity has a positive effect on loyalty.*

**H3b.**  *Place dependence has a positive effect on loyalty.*

**H3c.**  *Place identity has a positive mediating effect on the relationship between food involvement and loyalty.*

**H3d.**  *Place dependence has a positive mediating effect on the relationship between food involvement and loyalty.*

### 2.9. The Moderating Effect of Lifestyle

Consumer groups with different lifestyles significantly differ in socio-demographic, attitudinal, and behavioural aspects [87]. Tourism scholars have explored the influence of lifestyle on tourists’ attitudes and behaviours related to tourism. Lifestyles affect people’s behaviours in consuming tourism products or services [87]. Lifestyles are associated with vacation styles and impact satisfaction, loyalty, and word-of-mouth [78]. Similarly, food tourists are heterogenous [78]. Few attempts have been made to classify food tourists according to their levels of involvement and motivation [22,34]. Involvement and motivation are indicators of tourists’ lifestyles [88]. The degree of involvement is affected by one’s needs, values, and interests, which are indicators of lifestyle [39]. The attitudes, intentions, and planning behaviours of food tourists with different levels of involvement are distinctive [22]. Specifically, tourists with different lifestyles may have different perceptions and understandings of the relationship between food involvement, place identity, place dependence, and destination loyalty. For example, tourists with more adventurous lifestyles might be more interested in novel food and highlight the unique food experience in the destination [89]. In this case, food involvement could exert a higher impact on place identity and place dependence and lead to more loyalty. Based on the previous discussions, we proposed the following hypotheses:

**H4a.**  *Lifestyle moderates the relationship between food involvement and place identity.*

**H4b.**  *Lifestyle moderates the relationship between food involvement and place dependence.*

**H5a.**  *Lifestyle moderates the relationship between place identity and loyalty.*

**H5b.**  *Lifestyle moderates the relationship between place dependence and loyalty.*

**Figure 1:**
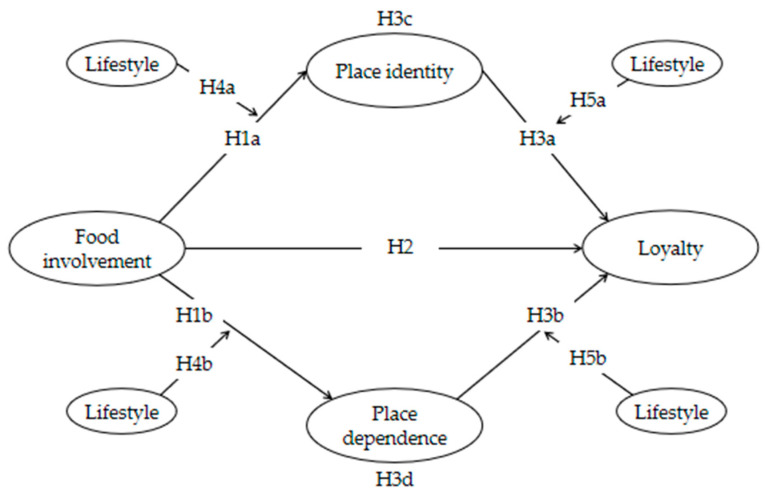
Research model.

This study proposes a direct relationship between food involvement and loyalty and an indirect relationship mediated by place identity or place dependence. Regarding the involvement–attachment–loyalty link, the first and second stages of the moderating effects of lifestyle are also proposed (see Figure 1).

## 3. Methods

### 3.1. Questionnaire and Pilot Test

The questionnaire used in this study included five parts. The first part catered for tourists’ food involvement with six items [40,43]. The second part focused on tourists’ attachment to the destination, including four items for place identity and four items for place dependence [47,48,90]. The third part concerned tourists’ loyalty, with three items on willingness to recommend and revisit a place [47,91]. The fourth part revolved around tourists’ lifestyles, with ten items on health, family, sports, work, travel, adventure, culture, novelty, leisure time, and environmental awareness [77]. The fifth part gathered demographic information. A 5-point Likert scale was adopted for the scales of food involvement, place attachment, and loyalty (1 for “strongly disagree” and 5 for “strongly agree”). Lifestyle was also measured using a 5-point Likert scale, with 1 representing “very unimportant” and 5 representing “very important”.

The pilot survey was conducted in January 2021 using the convenience sampling method. The year-1 graduate students who were currently enrolled in Macau University of Science and Technology were invited to participate in survey. The questionnaire (Appendix A) was generated on a survey platform called Questionnaire Star, and then distributed online via graduate students’ WECHAT group. A screening question on the experience of visiting the study site (Shunde) was used. A total of 118 responses were collected. The overall reliability coefficient of Cronbach’s α was 0.902, and the reliability of each scale was greater than 0.8, indicating a good internal consistency [92]. 

### 3.2. Data Collection

The primary survey was conducted in March 2021. We selected three representative restaurants in Shunde to distribute the questionnaires. This approach suggests that the purposive sampling method was adopted. As a non-probability sampling technique, purposive sampling suggests that the researcher selects and determines the research subject according to the aims and objectives of a given study. Although this sampling method may lead to bias, purposive sampling can allow each respondent to “provide unique and rich information of value to the study” (p. 4) and maximise the utilisation of available resources [93]. We selected restaurants at the following three economic levels: high, middle, and low. This approach can address all consumption levels and select different catering categories, so that catering groups with different food preferences can be explored. Eight restaurants were selected for the questionnaire distribution. The surveyors were made up of one researcher and two volunteers. Volunteers were trained in advance to become familiar with conducting the survey. The survey was conducted on three weekends of March near the exit of restaurants when visitors come out after dinning. Prior to filling out the questionnaire, respondents were asked whether they are tourists to ensure the qualification of respondents. To encourage tourists to participate in this survey, surveyors prepared key chains for respondents as souvenirs. A total of 482 responses were collected. We retained 459 valid questionnaires for the data analysis after removing incomplete and invalid responses.

### 3.3. Demographic Profile

The profile of the respondents is presented in Table 1. Most were female and more than half were younger than 30 years old. More than one-third were enterprise employees, and students also accounted for one-third of the individuals. More than three-quarters of respondents held a degree. Most of them (65%) had a monthly income of less than CNY 10,000. More than half were from markets beyond the geographical range of Guangdong province.

## 4. Results

### 4.1. The Measurement Model

**Figure 2:**
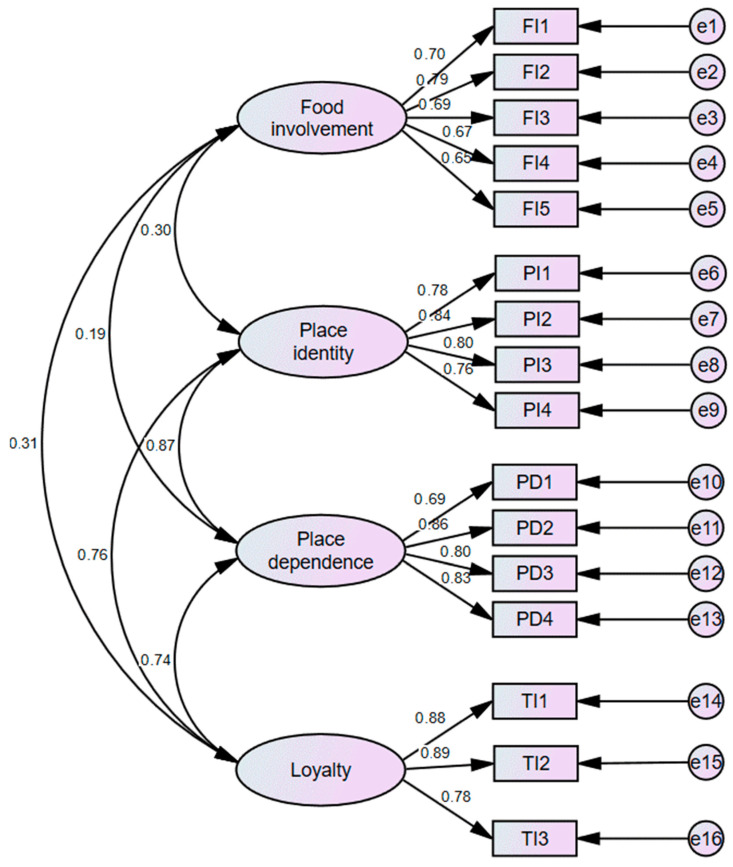
The measurement model.

The values of Cronbach’s α of the sales of food involvement, place attachment, and loyalty were all higher than 0.7, which indicates acceptable results. The CITC value of FI6 was lower than 0.4, which shows a weaker relationship with the other items. Therefore, it was eliminated from the list. Prior to the confirmatory factor analysis (CFA), KMO (Kaiser–Meyer–Olin) and Bartlett’s examinations were executed to ensure the readiness for factor analysis. The KMO value of 0.920 and the significance of Bartlett’s examination close to 0.000 manifest the rationality of factor analysis. AMOS21.0 was used to conduct the CFA analysis. The specific results are presented in Figure 2 and Table 2. The composite reliability (CR) values are required to be above 0.7, and the average extraction variance (AVE) values should be above 0.5 to indicate that the model had a high reliability and good convergence validity properties [95]. The standard factor loading of all items in the model was more significant than 0.6, the CR of each dimension was greater than 0.8, and the AVE was greater than 0.6, except for FI1 with 0.492. The model had good convergent validity. To test the model’s discriminant validity, according to Gaski and Nevin [96], the correlation value between each variable has to be less than 1. The tested results showing the confidence intervals of the variables do not include 1, which means the collinearity of variables does not exist. Therefore, the discriminant validity was established.

### 4.2. Path Analysis

The results of the CFA indicate that the measurement model adequately fits the data. The ratio of x^2^ to the degrees of freedom (x^2^/df = 2.38) and other commonly used goodness-of-fit indices (CFI = 0.97, IFI = 0.97, and RMSEA = 0.06) were in line with the established criteria (1 < x^2^/df < 3, CFI > 0.90, NNFI > 0.90, and RMSEA ≤ 0.08) [97,98].

**Figure 3:**
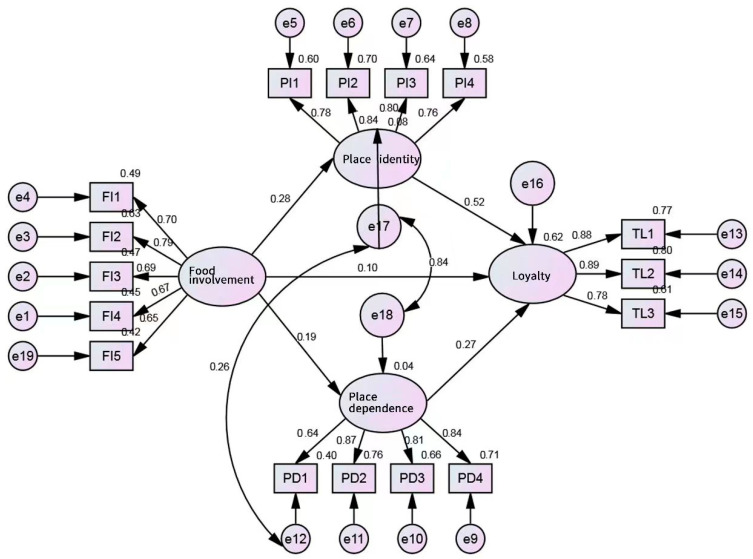
Path analysis results.

As indicated in Figure 3, the path coefficient estimate between food involvement and place dependence is 0.19 (*p* < 0.001), showing a positive relationship between these two variables. The path coefficient estimate between food involvement and place identity of 0.28 (*p* < 0.001) and the coefficient estimate between food involvement and place loyalty of 0.10 (*p* < 0.05) both verify the positive relationships between these variables.

### 4.3. Mediating Effect Test 

The mediating effect was tested by following the steps proposed by Baron and Kenny [99]. To test for mediation, the following regression equations were tested: first, regressing the mediator on the independent variable; second, regressing the dependent variable on the independent variable; and third, regressing the dependent variable on both the independent variable and on the mediator. Table 3 presents the hypothesis test results of H1a, H1b, H2, H3a, H3b, H3c, and H3d.

### 4.4. Cluster Analysis and Moderating Effects of Lifestyle

Prior to the principal component analysis (PCA), KMO (Kaiser–Meyer–Olin) and Bartlett’s examinations were conducted to ensure the suitability of proceeding to the factor analysis step. The results of the KMO and Bartlett’s examinations satisfy this requirement. The principal component analysis (PCA) was used to extract factors. Normally, the extraction of factors is based on the rule of an eigenvalue greater than 1, while the cumulative variance explained more than 60% [100]. However, in this case, the cumulative variance explained is 51.04% if we only extract factors with an eigenvalue greater than 1. To make the cluster of tourists more meaningful, five factors were extracted, with the cumulative variance explained at 74.9%. 

Through the principal component analysis and dendrogram of clustering, the lifestyle types of tourists can be classified into five categories. First, the traditional type (25.9%), who values family, health, and safety, and dislikes adventurous and novel experiences. Second, the motivated type (29.4%), who values work and sports and is active in terms of life, work, and sports. They hope to discover a travel destination where they can relax their minds and bodies. Third, the trendy type (18.5%), who values travel and novel experiences, but dislikes sports. They have high expectations of their destinations. Fourth, the literary–artistic type (9.1%), who pays attention to cultural, leisure, and environmental issues. Fifth, the adventure type (16.9%), who values adventure and prefers to seek excitement and challenges during travels. Prior to performing the moderating test, the following hypotheses were proposed:

**H4a(1).**  *Traditional type lifestyle moderates the relationship between food involvement and place identity.*

**H4a(2).**  *Motivated type lifestyle moderates the relationship between food involvement and place identity.*

**H4a(3).**  *Trendy type lifestyle moderates the relationship between food involvement and place identity.*

**H4a(4).**  *Literary–artistic type lifestyle moderates the relationship between food involvement and place identity.*

**H4a(5).**  *Adventure type lifestyle moderates the relationship between food involvement and place identity.*

**H4b(1).**  *Traditional type lifestyle moderates the relationship between food involvement and place dependence.*

**H4b(2).**  *Motivated type lifestyle moderates the relationship between food involvement and place dependence.*

**H4b(3).**  *Trendy type lifestyle moderates the relationship between food involvement and place dependence.*

**H4b(4).**  *Literary–artistic type lifestyle moderates the relationship between food involvement and place dependence.*

**H4b(5).**  *Adventure type lifestyle moderates the relationship between food involvement and place dependence.*

**H5a(1).**  *Traditional type lifestyle moderates the relationship between place identity and loyalty.*

**H5a(2).**  *Motivated type lifestyle moderates the relationship between place identity and loyalty.*

**H5a(3).**  *Trendy type lifestyle moderates the relationship between place identity and loyalty.*

**H5a(4).**  *Literary–artistic type lifestyle moderates the relationship between place identity and loyalty.*

**H5a(5).**  *Adventure type lifestyle moderates the relationship between place identity and loyalty.*

**H5b(1).**  *Traditional type lifestyle moderates the relationship between place dependence and loyalty.*

**H5b(2).**  *Motivated type lifestyle moderates the relationship between place dependence and loyalty.*

**H5b(3).**  *Trendy type lifestyle moderates the relationship between place dependence and loyalty.*

**H5b(4).**  *Literary–artistic type lifestyle moderates the relationship between place dependence and loyalty.*

**H5b(5).**  *Adventure type lifestyle moderates the relationship between place dependence and loyalty.*

This study used Stata13.0 to test the moderating effect of lifestyle. If the relationship between variables Y and X was a function of variable M, then M was referred to as the moderating variable [101]. The typical model is Y = aM + bM + cXM + e. A hierarchical moderated regression analysis was conducted to observe if the interaction between each of the two variables was statistically significant [26]. For instance, if the regression coefficient of lifestyle type 2 × food involvement and place identity was positive (*p* < 0.001), it demonstrated that lifestyle type 2 can enhance the relationship between food involvement and place identity. The test results are presented in Table 4, with the significant lifestyle types displayed in bold.

Regarding the link between food involvement and place identity, certain lifestyles (motivated, literary–artistic, and adventure) can strengthen the link. H4a was thus partially supported. In relation to the impact of food involvement on place dependence, literary–artistic lifestyle tourists can enhance the influence of food involvement on place dependence—therefore, H4b was partially supported. In relation to the effect of place identity on loyalty, trendy lifestyle tourists can strengthen the influence of place identity on loyalty—therefore, H5a was partially supported. As a consequence of place dependence on loyalty, trendy lifestyle tourists can also reinforce the effect of place dependence on loyalty—therefore, H5b was partially supported.

## 5. Discussion

This study determines that food involvement positively impacts place attachment and that the impact on place identity is greater than the impact on place dependence, indicating that tourists’ food involvement in their destination is linked to an emotional rather than a functional attachment. Gross and Brown [38] also revealed that the involvement of food and beverage positively affects the tourists’ place attachment; however, they observed that the effects of food involvement on place identity and place dependence are not significantly different.

The results also show that food involvement positively affects loyalty, suggesting that tourists who are involved with food are more likely to produce a positive evaluation of the place and a willingness to revisit it. This is consistent with the results of a study conducted by Kim et al. [43] that showed a significant positive correlation between tourist food involvement and tourist loyalty. This study also indicated that the influence of food involvement on loyalty is small, and the reasons for tourists to become loyal should include many other factors, which were also mentioned in previous studies [42,43].

This study reveals the indirect effect of food involvement on loyalty through place identity or place dependence. The influence of food involvement on loyalty through place identity is greater than the direct effect of food involvement on loyalty. However, the indirect effect through place dependence is the opposite. This may be because the mediating effect of place identity is greater than that of place dependence. Place identity is the most important dimension of place attachment [79] and is more significant than place dependence in influencing tourists’ loyalty [48]. 

Overall, the results are consistent with the S-O-R theory. The theory emphasizes the mediating role of the organism in affecting reactions and behaviour. Therefore, the direct influence of stimuli on the response may be limited, as reflected in this study by the small direct influence of food involvement on loyalty. However, when place attachment was introduced and examined as a mediator, it exhibited a more significant influence on loyalty. Furthermore, the greater effect of place identity compared to place dependence aligns with the original assumption of the S-O-R theory, indicating that emotions are important antecedents of response [25].

Moreover, this study confirms the moderating role of lifestyle. The results indicate that tourists’ different lifestyles play important roles in interacting with food involvement, thereby influencing the travel outcome. In this study, tourists are categorized into various types, with each type exhibiting unique characteristics. While some tourists prioritize novelty and freshness in their lives, others tend to value familiarity and tradition. These individual traits guide tourists in determining the degree of importance and interest they place on food. Therefore, lifestyle can interact with food involvement to impact the emotional bond that tourists form with their travel destination. Similarly, tourists with different lifestyles have their own preferred activities in a travel destination. As a result, the touristic experiences formed through these preferred activities contribute to strengthening the emotional connection between the tourists and the destination. This, in turn, has an impact on destination loyalty. In this scenario, the moderating role of lifestyle between place attachment and loyalty becomes apparent. 

Additionally, the confirmed moderating role of lifestyle also indicates a moderated mediating effect. Specifically, the influence of food involvement on loyalty via place identity would differ depending on the moderating role of lifestyle. Similarly, the influence of food involvement on loyalty via place dependence would also differ depending on the moderating role of lifestyle. These findings suggest that the interaction effect between lifestyle and food involvement can have a profound influence not only on place attachment, but also on behavioural outcomes such as loyalty. Likewise, the moderated mediating effect can be observed when lifestyle interacts with place attachment, indirectly affecting loyalty. Therefore, when tourists exhibit different lifestyles and place varying levels of importance on food, it not only impacts their place attachment, but also has an indirect effect on loyalty.

Specifically, this study shows that different types of tourists have significantly different emotional connections with a destination. The possible reasons for these results are the following: motivated tourists attach importance to work and sports, so they hope to stay away from the noise and achieve a complete relaxation of the body and mind, and the motivation of food tourism includes an escape from reality [27]. As a result, motivated travellers who perceive food as necessary in their travels are more likely to develop an emotional attachment to the destination. 

While adventurous tourists enjoy excitement, the unique food experience of the tourist destination may strengthen the attachment of adventurous tourists to the destination. Previous studies have also confirmed that tourists who are highly receptive to novel foods positively impact destination food consumption and can enhance tourists’ food satisfaction [102], while satisfaction positively affects both dimensions of place attachment [47]. 

Cultural tourists value culture, leisure time, and environmental awareness, and tourists’ local food consumption behaviour is considered a “symbolic” social experience, and tourists can experience local culture by tasting the local food [103]. Therefore, cultural tourists who believe food is essential in their travels are more likely to develop an emotional and functional attachment to the destination. 

Trendy lifestyle tourists pay more attention to travel and novel experiences and enjoy travelling more than other types of tourists. Therefore, after developing an attachment to the destination, under the condition that the other influencing factors remain unchanged, trendy lifestyle tourists are more likely to present loyalty to the destination than other types of tourists. As the moderating roles of lifestyle in food involvement, place attachment, and loyalty are still new attempts, there are still shortcomings in understanding the moderating role of lifestyle, but this opens up new ideas for understanding tourists’ behaviours.

The contribution of this study to existing tourism knowledge is three-fold. First, it confirms the significance of food involvement in influencing tourists’ place attachment. Although the influence of food and alcohol involvement on tourists’ place attachment was confirmed [38], no scholars were involved in this research since then. This study demonstrates the significance of food involvement in influencing tourist loyalty. Whether food involvement can affect tourists’ loyalty is yet to be confirmed in the literature. Chen and Huang [104] proposed that the reason for the difference in their research results compared to those of Kim et al. [43] is that the nationalities of the respondents are different. This study does not agree with this claim but believes that different investigation backgrounds cause the difference in the results. In the context of food tourism, food involvement has a positive impact on tourist loyalty and vice versa.

Secondly, from the perspective of the S-O-R theory, this study provides valuable insights. It simultaneously examines place identity, a concept that is more oriented towards emotions, and place dependency, a concept that is more focused on functionality and cognition. The results indicate that place identity has a greater mediating effect on loyalty compared to place dependency. While some scholars have argued for the inclusion of cognitive elements in the S-O-R theory to broaden its applicability [105], there have been limited studies comparing and discussing which components (emotional and cognitive) have a more significant influence on human reactions. Therefore, this study offers empirical evidence and identifies that emotional elements have a greater impact on human behaviour in the context of food tourism.

Third, the introduction of lifestyle as a moderating variable enriches the relevant results of food tourism research. Lifestyle is the most effective segmentation tool in the field of marketing [106]. This study divides tourists into five types according to lifestyle, and the moderating effects of different types of tourists on food involvement, place attachment, and loyalty are investigated. This study’s results verify that the influence of different types of tourists on the relationship between variables is significantly different. Such research opens up new ideas for the field of tourism. 

## 6. Implications and Limitations

### 6.1. Implications

From a managerial perspective, this study also provides significant practical implications for destinations’ food tourism development. The results show that tourists’ food involvement affects destination loyalty through place identity and place dependence. Therefore, for destinations intending to develop food tourism and apply local food as a useful attraction, food-related activities in the tourism destination should be diversified and enhanced. For example, food festivals or culinary classes are desirable activities to offer in the tourism destination [15]. Successful examples include the Thai culinary classes in Thailand and the Food and Wine Festival in Melbourne, Australia. Moreover, destinations can design gastronomic day tours for the tourists with a high food involvement to participate in, such as the Market Street Food Tour in Hoi An, Vietnam and the Sunset food tour in Rome, Italy [107]. Providing information about local food culture and traditional cuisine to tourists with a high food involvement can enrich their food experience.

On the other hand, tourists’ lifestyles were confirmed as the meaningful moderator in this study. Such results provide practical information to destination marketers and food tourism operators to develop marketing strategies tailored to suit different segments. Overall, tourists with motivated lifestyles enhance the relationship between food involvement and place identity. For this segment, a relaxing mindset and enjoying life are essential. Therefore, the destination can provide the food experience in a pleasant environment, peaceful atmosphere, and calming vibe. In this case, a restaurant located on the mountain and seaside with wonderful scenic views can easily help motivate tourists to achieve a relaxing mindset [108]. 

For tourists with trendy lifestyles, destination markers could highlight the novel and fun experience of the destination food provision. For example, providing novel types of food or ingredients and targeting this segment are valuable strategies [93]. Meanwhile, new culinary skills, table manners, and unfamiliar tastes are critical factors that highlight destination food promotion [109]. Specifically, this study encourages destinations to be more innovative and flexible in their food offerings. The coffee apartment concept in Vietnam is a successful example that turns the local coffee culture into a fashionable and exotic experience for their tourists. The stylish décor and the lively atmosphere make it become a well-known food-related tourist spot. 

Regarding the literary–artistic segments, destination marketers should focus more on the traditional, cultural, and environmental issues of the destination food provision. Therefore, destination restaurants can highlight traditional meals with in-depth local culture [110]. Moreover, destination catering providers can promote local ingredients in their food to enhance the eco-friendly image by reducing their carbon footprint [111]. The unique Macanese food in Macau is an ideal example, which is not only an intangible cultural heritage, but also a must-try traditional cuisine among tourists. For tourists with an adventurous lifestyle, a helpful marketing strategy lies in the introduction of the unusual or unfamiliar foods of a travel destination. Although tourists do not generally embrace unfamiliar types of food, it could fulfil the needs of tourists with an adventurous lifestyle [112]. For instance, sheepshead is a traditional meal in Norway. For some eaters, this meal can provide an enjoyable and adventurous experience and imply a feeling of courage [113]. Similarly, other examples include the consumption of various insects in some Asian countries or fish eyeballs in Japan. Marketing strategies such as promoting unique and unfamiliar food in travel destinations could attract tourists with adventurous lifestyles who actively seek novel and daring culinary experiences.

### 6.2. Limitations

Four limitations of this research are identified. First, the respondents were young or middle-aged. Therefore, the results of this study may not be applicable to all age groups. Second, this study focused on the food experience during the trip, thus neglecting relevant food tourism experiences at the pre-trip or post-trip stages [104]. Pre-trip tourists may search or collect tourism and destination information through various channels. At the post-trip stage, tourists may recall their memorable experience of destination food, share the local food products they bought with others, and share their experiences on social media. Food tourism experience prior to or following the trip can be a direction for future research. Third, due to the nature of place dependence often reflecting the importance of the place in achieving one’s functional activities, it would be better to ask more specific questions related to food activities in Shunde rather than about the place itself. Fourth, this study investigated the mediating effect of place attachment and the moderating effect of lifestyle. However, the reliance on statistical approaches limited the comprehensive explanation of the conceptual model. To gain a more holistic understanding, future research is encouraged to explore alternative statistical techniques that can examine the moderated mediating effect of lifestyle, thus providing new and valuable insights into the role of lifestyle as a moderator. 

## Figures and Tables

**Table 1:** Profile of respondents.

	Frequency	Percent
**Gender**		
Male	176	38.30%
Female	283	61.70%
**Age**		
≤18	13	2.80%
19–29	250	54.46%
30–39	124	27.01%
40–49	63	13.73%
50–59	9	2.00%
**Education**		
High school or below	27	5.90%
College	80	17.40%
Bachelor’s	296	64.50%
Master’s or above	56	12.20%
**Monthly income** *		
≤¥3000	144	31.40%
¥3000–4999	64	13.90%
¥5000–6999	92	20.00%
¥7000–8999	52	11.30%
≥¥9000	107	23.30%
**Profession**		
Student	157	34.20%
Staff of government agencies/institutions	70	15.30%
Company/business staff	173	37.70%
Private owner/self-employed	22	4.80%
Housewife	5	1.10%
Soldier	3	0.70%
Freelancer	12	2.60%
Retirees	2	0.40%
Others	15	3.30%
**Residence**		
Guangdong (not including Shunde)	219	47.70%
Hong Kong	10	2.20%
Macau	8	1.70%
Beijing	50	10.89%
Tianjin	30	6.53%
Jiangsu Province	60	13.07%
Hunan Province	82	17.86%

* Note: monthly income categorization is based on Annual Report on China Household Income Distribution 2021 [94].

**Table 2:** Confirmatory factor analysis results.

Factor	Item	Factor Loading	CR	AVE
Food involvement	FI1	0.70	0.828	0.492
	FI2	0.79		
	FI3	0.69		
	FI4	0.67		
	FI5	0.65		
Place identity	PI1	0.78	0.873	0.633
	PI2	0.84		
	PI3	0.80		
	PI4	0.76		
Place dependence	PD1	0.69	0.874	0.636
	PD2	0.86		
	PD3	0.80		
	PD4	0.83		
Loyalty	TL1	0.88	0.887	0.725
	TL2	0.89		
	TL3	0.78		

FI1: Tasting food is the most expected activity; FI2: choosing local food is critical; FI3: eagerness to try local food; FI4: caring about local food; FI5: sharing food experience with others; PI1: Shunde is a special place; PI2: I like Shunde very much; PI3: it is meaningful to travel in Shunde; PI4: have a strong sense of belonging to Shunde; PD1: Shunde is irreplaceable; PD2: I like Shunde more than other destinations; PD3: Shunde provides me with more leisure and enjoyment; PD4: I am more satisfied with Shunde than other destinations; TL1: I will promote Shunde with positive information; TL2: I will recommend Sunde to my relatives and friends; TL3: I will visit Shunde again.

**Table 3:** Results of hypotheses test.

Hypothesis	Path	Direct Effect	Indirect Effect	Total Effect	Test Result
H1a	Food involvement → place identity	0.348 ***	——	——	Supported
H1b	Food involvement → place dependence	0.255 ***	——	——	Supported
H2	Food involvement → loyalty	0.124 **	——	——	Supported
/	Place identity → loyalty	0.518 ***	——	——	Supported
/	Place dependence → loyalty	0.244 ***	——	——	Supported
H3a and H3c	Food involvement → Place dependence → loyalty	0.101 ***	0.218 ***	0.319 ***	Supported
H3b and H3d	Food involvement → place identity → loyalty	0.167 ***	0.152 ***	0.319 ***	Supported

Note: *** *p* < 0.01, ** *p* < 0.05.

**Table 4:** The moderating effect of lifestyle test results.

Hypothesis	Path	Traditional	Motivated	Trendy	Artistic	Adventure
H4a	Food involvement → place identity	−0.0909 (0.0581)	**0.159** ** (0.0648)	0.0143 (0.0654)	**0.168** *** (0.0596)	**0.136** * (0.0718)
H4b	Food involvement → place dependence	**0.0757** *** (0.0285)	0.0234 (0.0437)	0.0178 (0.0352)	0.159 ** **(0.0648)**	0.0143 (0.0654)
H5a	Place identity → loyalty	0.00843 (0.0307)	−0.0358 (0.0620)	0.0757 *** **(0.0285)**	0.0234 (0.0437)	0.0178 (0.0352)
H5b	Place dependence → loyalty	0.00892 (0.0319)	−0.0562 (0.0401)	**0.0607** * (0.0366)	−0.0562 (0.0371)	−0.00462 (0.0353)

Note: *** *p* < 0.001, ** *p* < 0.01, * *p* < 0.05.

## Data Availability

The data used to support the findings of this study are available from the corresponding authors upon request.

## Appendix A. Questionnaire

Dear Sir/Madam,

Hello! We are the graduate students of the Faculty of Hospitality and Tourism Management at Macau University of Science and Technology and conducting research on the relationships among tourists’ food involvement, place attachment, lifestyle and tourist loyalty. The aim is to understand whether tourists’ involvement in Shunde’s local cuisine will affect their place attachment and loyalty, and whether the impact on place attachment and loyalty varies depending on the tourists’ lifestyle. Thank you for your help and contributions to our academic investigation. This survey is filled in anonymously and will not be announced to the public. The information provided is in confidential and only for academic purposes. Thank you!

Screening question:

Are you a tourist (not local resident) currently visiting Shunde? Yes□ No□ If you answer yes, please proceed to our survey below and circle the number which you think is the most appropriate. If you answer no, please return the questionnaire to us. Thanks.

**Item**	**Strongly Disagree**	**Agree**	**Neutral**	**Disagree**	**Strongly Disagree**
**Food involvement**					
When I travel, what I look forward to is the food.	5	4	3	2	1
When I travel, local food choices are important to me.	5	4	3	2	1
When I travel, I want to try local food.	5	4	3	2	1
When I travel, I care about the taste of local food.	5	4	3	2	1
I love talking to my friends about my food experiences while traveling.	5	4	3	2	1
I personally make most or all of the decisions when it comes to food choices for travel.	5	4	3	2	1
**Place attachment**					
Shunde is a special place for me.	5	4	3	2	1
I like Shunde very much.	5	4	3	2	1
The travel experience in Shunde means a lot to me.	5	4	3	2	1
I have a strong sense of belonging to Shunde.	5	4	3	2	1
Shunde is irreplaceable.	5	4	3	2	1
I prefer Shunde compared with other tourist destinations.	5	4	3	2	1
Shunde provides me with better leisure and enjoyment compared to other tourist destinations.	5	4	3	2	1
I am more satisfied with Shunde than other destinations.	5	4	3	2	1
**Tourist loyalty**					
I will spread positive information about traveling to Shunde to others.	5	4	3	2	1
I will recommend to my relatives and friends to travel in Shunde.	5	4	3	2	1
I would like to travel to Shunde again in the future.	5	4	3	2	1
**Lifestyle**					
How important are the following life factors in your life?	Very important	Important	Natural	Unimportant	Very unimportant
Healthy	5	4	3	2	1
Family	5	4	3	2	1
Sports	5	4	3	2	1
Work	5	4	3	2	1
Travel	5	4	3	2	1
Adventure	5	4	3	2	1
Culture	5	4	3	2	1
Novel	5	4	3	2	1
Leisure time	5	4	3	2	1
Environmental awareness	5	4	3	2	1
**Personal information**					
Gender: male□ female□					
Age: ≤18□ 19–29□ 30–30□ 40–49□ 50–59□ ≥60□					
Profession: student□ government agency/institution staff□ company/corporate staff□ private owner/self-employed housewife□ soldier□ freelancer□ retirees□ other□					
Monthly income: ≤3000□ 3000–4999□ 5000–6999□ 7000–8999□ ≥9000□					
Education level: high school graduated or lower□ associate degree/technical school graduate□ bachelor’s degree□ master’s degree or higher□					
Residency: Guangdong Province (excluding Shunde)□ Hong Kong□ Macao□ other regions□					
